# The Georgia Medical Society and the Tariff

**Published:** 1888-03

**Authors:** 


### THE GEORGIA MEDICAL SOCIETY AND THE TARIFF.

The circular of the Secretary of the Georgia Medical Society, printed on another page of this journal, calls the attention of the medical profession to the onerous exaction of the existing tariff laws in the high duties on medicines and surgical instruments, and solicits the co-operation of all persons interested in an attempt to induce Congress to mitigate or abolish the evil.

This seems to be an auspicious time for this attempt, as Congress is now considering a general revision of the revenue laws, in which it is possible some relief in the direction indicated may be obtained, while it wouid be most unlikely that it could be secured by a special enactment, as was done in the case of the duty on quinine.

By the tariff law the four or five thousand articles, upon which duties are levied, are classified in such manner that those referred to in the circular fall in different classes, and pay different rates of duty, some of them very high, others at a more reasonable rate, so that it is difficult to ascertain the exact amount of tax paid by the consumer upon each individual article. The general average paid upon all dutiable merchandise is about forty-five per cent.; some of those included under the name " medicines and appliances " in the circular pay as high as one or two hundred per cent.

" Surgical instruments " bear no specific rate of duty under that name ; they are comprised under the general term, "cutlery," and pay about the average duty, forty-five per cent. This simple statement conceals, or at least does not reveal, the enormity of this tax—a plain illustration will better serve that purpose.

A Georgia doctor wishes to buy one hundred dollars' worth of surgical instruments made in London or Paris ; the importer has to pay forty-five dollars tax on them at the port of entry ; the cost to him is, therefore, one hundred and forty-five dollars, on which sum he is entitled to charge reasonable profit, together with insurance, freight, risk from breakage, rust, etc. All these would amount to not less than fifty per cent. upon the cost and duty, making seventy-five dollars and fifty cents, which, added to one hundred and forty-five, gives two hundred and seventeen dollars and fifty cents as the cost to the Georgia doctor in New York.

Nor can he escape this exaction by buying his instruments from a home manufacturer. That worthy citizen would demand a like sum for a like quality of goods. In the one case part of the money wrung from the purchaser would go to swell the bursting treasury of the United States Government ; in the other to distend the bloated pockets of the juvenile home industrial.

The objects sought by the time-honored Georgia Medical Society have our warmest sympathy and cordial coöperation. They present and emphasize one single point on which the Octopian protective policy infringes upon the interests of our guild. In seeking to free the millions of our suffering fellow tax-payers from some of the iniquities and injustice of that policy, we hope that Congress will not forget or fail to remove our special grievance.

### ITEMS.

A MOVEMENT is on foot to establish a respectable hospital in this city. The result cannot be foretold.

DR. D. J. CAIN, a distinguished physician of Asheville, N. C., and formerly of Charleston, has retired from practice, and removed to Aiken, S. C.

THE commencement exercises of the Atlanta Medical College will occur at DeGive's Opera House, March 1st. The class is larger than for several years past.

DR. HENRY L. MILLER, a prominent physician of Overton, Texas, died January 23d, 1888, after a brief illness of only forty-eight hours, of congestion of the bowels.

DR. S. F. STARLEY, Ex-President of the Texas State Medical Association, died at his home in Tyler, Texas, December 19, 1887.

THE Medical Association of Georgia will meet in Rome, April 18, 1888, and continue in session three days. Every doctor in the State should attend and become a member.

DRS. A. W. GRIGGS, of West Point; A. G. Whitehead, of Waynesboro, and Thos. O. Powell, of Milledgeville, were in the city recently and gave THE JOURNAL a call.

DR. H. B. TATE (A. M. C., 1886), of Union, S. C., was married January 1st, 1888, to Miss Amanda Gondelock, of South Carolina.

IN an extensive practice, covering seventeen years, Dr. B. W. Mason, of Sheridan, Ark., has only used the forceps in one case and has never lost a mother or child. A good record.

WE recently had a pleasant call from Mr. E. B. Treat, the publisher at 771 Broadway, New York. Mr. Treat is issuing "Medical Classics," a series of books on various subjects, by noted American specialists. Five volumes have been issued so far, and after examination we can commend them to our readers.

FRANKLIN LEONARD POPE, an authority in electrical affairs, will contribute to the March number of *Scribner's Magazine* a paper on the "Electric Motor and its Applications," which is a complete account in brief compass of the origin and development of the use of electricity as a motive power. It will be fully illustrated.

LEA BROTHERS & CO., of Philadelphia, will shortly publish a Clinical Atlas of Venereal and Skin Diseases, including Diagnosis, Prognosis and Treatment, by Professor Robert W. Taylor, M. D., formerly President of the American Dermatological Association, and joint author of Bumstead & Taylor's Pathology and Treatment of Venereal Diseases. The work will be issued in eight parts, aggregating 58 large folio chromo-lithographic plates, measuring 14 x 18 inches, and containing about 200 figures, many of them life-size, executed with the utmost faithfulness and beauty of detail. These plates will delineate typical cases from the practice of the author, and selections from the entire literature of Europe, including among others the works of Cullerier, Fox, Fournier, Hebra, Hutchinson, Kaposi, Neumann and Ricord. The text will deal chiefly with the practical aspects of the subjects, and will be illustrated with a series of unusually large engravings, executed especially for this work, and drawn principally from original matter in the possession of the author.

THE March *Century* will contain the story of "Colonel Rose's Tunnel at Libby Prison," told by one of the one hundred and nine Union officers who escaped on the night of February 9, 1864. The successful construction of this tunnel—dug from a dark corner of the cellar of the prison, through fifty feet of solid earth, the only tools being two broken chisels and a wooden spittoon in which to carry out the dirt—was one of the most remarkable incidents of the war. Colonel Rose, to whose indomitable will and perseverance the success of the scheme was due, is now a captain in the 16th United States Infantry, and of the fourteen men who assisted him in digging the tunnel, eleven are still living. The narrative in the March *Century*, which is illustrated, forms one of the untechnical papers supplementing the war series, and it is said to be one of the most romantic records that the *Century* has ever printed.

TASTELESS PREPARATION OF CASCARA SAGRADA (RHAMNUS PURSHIANA).—Recent investigation of the constituents of Cascara sagrada has led to the discovery of new principles and facts of great importance pharmaceutically and therapeutically.

The chief objection to Cascara sagrada heretofore has been its inherent bitterness. In the light of recent researches, tasteless preparations of this drug, highly efficacious medicinally, are now to be had.

These discoveries mark a distinct advance in pharmacal attainment and in the therapeutics of chronic constipation, since this remedy can now be much more generally and persistently administered, and its well-known tonic laxative action obtained without the drawbacks which seemed formerly inseparable from its employment.

The facts disclosed concerning this remedy deserve more than a passing notice, especially since they indicate the existence of principles and modes of action extending far beyond the subject indicated, and are well worth the close attention of the thoughtful and scientific physician. A valuable contribution to the knowledge of the chemical constitution of this drug appeared in the *American Journal of Pharmacy*, for February, 1887, which makes it possible not only to obtain a true interpretation of the various clinical observations, but clears up apparent anomalies and also indicates the reasons for observed effects, which have lately been disputed, but now admit of no further question or misunderstanding.

Among the discoveries referred to in this valuable paper of especial interest to the physician is the influenee of a class of vegetable ferments, and their recognition as the causes of various abnormal conditions, such as colic, vomiting, nausea, diarrhœa and dysentery which occasionally attend the administration of certain drugs.

It appears that Frangula bark, when fresh, contains such a ferment in excessive quantities and is, therefore, unfit for use until the ferment has exhausted itself—the process usually occupying several years. It also appears that Cascara contains some of this principle, and this fact will account for the occasional untoward effects of the drug, which have been observed as consequent on the employment of a number of its preparations heretofore in the market. These effects are, therefore, not due, as has been supposed, to any idiosyncrasy on the part of the patient, or to the laxative or tonic constituents of the bark itself, but to a distinct objectionable principle, which once recognized can be rendered inoperative and harmless.

It has been reserved for Parke, Davis & Co., through their exhaustive investigations, to be the first to clearly recognize the principles involved, and by the application of such intelligent comprehension to formulate and adopt correct pharmaceutical processes and thus overcome all the difficulties heretofore existing. As a result of their investigations, they now offer to the medical profession a fluid extract, a solid extract and also a concentration, all of which (designated as "Formula of 1887") exhibit only the desirable laxative and tonic properties, and being free from this ferment are incapable of producing griping, nausea or any of the mal-effects above enumerated.

It appears that these ferments are distributed through a large number of vegetable substances, being not confined to unripe fruits only, but can also exist in the root, bark, leaf, or even in vegetable extracts, of which we have illustrations in various juices, liquid or inspissated. Of this latter class, aloes will serve for an example. A familiar illustration of an unaltered vegetable would be the cucumber, the green apple (familiar to the school-boy) and unripe fruit generally. In the case of the cucumber, experience has taught the means of removing this ferment by dialysis or osmosis. We sprinkle salt over it or surround it with a strong brine which provokes an outward flow of the fluid containing the ferment, with the result that the ferment is, to a large extent, removed, and thus rendered incapable of producing the same conditions in the stomach for which it was intended in the plant; that is, the creation of vegetable acids from other material previously existing, in the same manner that pepsin, likewise an unorganized and soluble ferment, provokes the solution of fibrin and albumen, forming peptone, or as diastase is capable of effecting the transformation of starch into soluble glucose and dextrin, both new bodies.

That these ferments all bear a direct quantitative proportion to the results accomplished has been practically recognized. We are promised a satisfactory indication of the sources of the acids formed in the plant, which will enable us to corroborate the statements that identical processes go on in the stomach when the ferment is permitted to exert its action there.

The physiological tests now being conducted at the laboratory of Parke, Davis & Co., with the different principles contained in the plant, cannot fail to demonstrate finally not only the superiority of Cascara itself to its former supposed competitor, Frangula, but also its comparative value as a laxative.

IDIOSYNCRASY WITH REGARD TO ANTIPYRIN—A WARNING.—A member of my family liable to migraine was attacked in the ordinary way a few days ago, and I administered for the first time a dose of 5 grains of antipyrin in powder with the following curious result: Five minutes after taking it, the "deadly sickness" which was previously present seemed to give way, and an "expanding sensation" was felt, rising from the stomach upwards. Almost immediately she sneezed violently for about twenty times running without pause. The face and eyes became deeply suffused; tears began to flow; quantities of mucus flowed from the nose; the breathing became hard and labored, accompanied by a feeling of suffocation; there was complete inability to lie down. A violent cough shortly came on, and large quantities of mucus were expectorated; at the same time there was very profuse sweating. After these phenomena had lasted for about half an hour, intense itching was felt on the insides of both thighs, and on examination there was found a thick outcrop of urticaria, which soon extended on to the abdomen. There was also a strong coppery taste in the mouth—not continuing, but coming on in violent bouts—and an equally strong smell of the same metallic nature, also intermittent. There was loud singing in the ears, which felt intensely congested. The pulse was quick and very full. After the symptoms had lasted about three-quarters of an hour from the commencement, they gradually disappeared, some tightness of the chest and running at the nose remaining for four or five hours longer. The sickness accompanying the migraine disappeared completely as soon as the drug had begun to work; the headache also disappeared for a time, but came back slightly about four hours afterwards. As antipyrin is now being so largely prescribed, I thought the above account might be of use to the readers of THE JOURNAL as showing the necessity for caution when prescribing it for a patient who has not previously taken it. —*W. Allen Sturge, in British Medical Journal.*

